# The validation of oxygen uptake efficiency slope in patients with stroke

**DOI:** 10.1097/MD.0000000000027384

**Published:** 2021-10-22

**Authors:** Sheng-Chieh Han, Tieh-Cheng Fu, Chih-Chin Hsu, Shu-Chun Huang, Hsin-Yu Lin, Jong-Shyan Wang

**Affiliations:** a Department of Physical Medicine and Rehabilitation, Chang Gung Memorial Hospital, Keelung, Taiwan; b Heart Failure Research Center, Division of Cardiology, Department of Internal Medicine, Chang Gung Memorial Hospital, Keelung, Taiwan; c School of Medicine, College of Medicine, Chang Gung University, Taoyuan, Taiwan; d Department of Physical Medicine and Rehabilitation, Chang Gung Memorial Hospital, Linkou, Taiwan; e Department of Gerontological and Long-term Care Business, School of Nursing, Fooyin University, Taiwan; f Graduate Institute of Rehabilitation Science, Department of Physical Therapy, College of Medicine, Chang Gung University, Taoyuan, Taiwan; g Research Center for Chinese Herbal Medicine, College of Human Ecology, Chang Gung University of Science and Technology, Taoyuan, Taiwan.

**Keywords:** cardiorespiratory fitness, exercise test, oxygen uptake efficiency slope, stroke, submaximal

## Abstract

To evaluate the real aerobic capacity is difficult due to impaired limbs function in stroke patients. Oxygen uptake efficiency slope (OUES) could represent the aerobic capacity in submaximal exercise test. Hence, we designed this observational study to investigate the application of the OUES for evaluating aerobic capacity in these patients.

Thirty-seven stroke patients were classified into 2 groups according to their Brunnstrom stage of affected lower limbs. Patients underwent cardiopulmonary exercise testing to assess cardiorespiratory fitness. Minute ventilation and oxygen consumption were measured, and OUES was calculated, compared with healthy reference values, and correlated with the peak oxygen consumption. The predictive validity of submaximal OUES was derived.

Study participants’ OUES (median 566.2 [IQR, 470.0-711.6]) was 60% of healthy reference values and correlated positively with the peak oxygen consumption (*r* = 0.835) (*P* < .01). The predictive validity of oxygen uptake efficiency slope at 50% of maximal exercise duration (OUES_50_) and oxygen uptake efficiency slope at 75% of maximal exercise duration (OUES_75_) for oxygen uptake efficiency slope at 100% of maximal exercise duration (OUES_100_) was 0.877 and 0.973, respectively (*P* < .01). The OUES_50_, OUES_75_, and OUES_100_ groups were not significantly different; agreement of submaximal and maximal OUES values was strong.

OUES is a valuable submaximal index for evaluating cardiorespiratory fitness in stroke patients. Moderate-to-high concurrent validity of this parameter with peak oxygen consumption and the high predictive validity of OUES_50_ and OUES_75_ for OUES_100_ suggest maximal exercise testing in stroke patients who cannot reach maximal exercise is unnecessary.

## 1 Introduction

There are several risk factors for stroke, including age, sex, obesity, smoking, hypertension, diabetes, hyperlipidemia, and atrial fibrillation.^[1]^ Exercising is beneficial for controlling many of these factors.^[2]^ Therefore, developing a valid measure to assess accurate cardiorespiratory fitness (CRF) in patients with stroke is important for designing an appropriate exercise prescription to reduce risk.

Currently, the most popular tool used to assess CRF is the cardiopulmonary exercise test (CPET), which measures peak oxygen consumption (VO_2peak_). For some, the longer-term consequences of stroke may include impaired motor function, reduced muscle strength and metabolic functioning, and reduced CRF.^[3,4]^ These patients have exercise intolerance and often have cognitive, motor, and sensory dysfunction, as well as gait disorders.^[5]^ Hence, patients with stroke may have difficulty meeting the criteria for maximal exercise and require submaximal exercise testing to assess their CRF.

However, for some reason, submaximal exercise testing may deviate from the criteria specified for maximal testing, including premature termination of the test, preset intensity, or discomfort reported by patients, rendering the adequate assessment of CRF in stroke patients difficult. This indicates the necessity for applying a specific objective and independent measure for submaximal testing. The oxygen uptake efficiency slope (OUES) is derived from the curve-linear relation between oxygen consumption (VO_2_) and the log transformation of the minute ventilation (minute ventilation [VE]), first introduced by Baba et al^[6]^ As an advantage of OUES, its calculation is independent of the CPET protocol and patient effort. Hence, OUES is a good surrogate, replacing the role of VO_2peak_, which represents the CRF in patients who cannot attain maximal effort during CPET.^[7]^

OUES has been extensively applied in various diseases, including obesity,^[8]^ heart,^[9,10]^ lung,^[11]^ and neurologic diseases.^[12]^ However, the validity of OUES has not been well demonstrated in stroke patients. Hence, this study was designed to investigate the impaired CRF of stroke patients using the traditional parameters from a CPET, to determine the OUES value of stroke patients, and to demonstrate the predictive validity of the OUES based on different levels of stroke-induced disability and different submaximal exercise duration to show whether the OUES from a submaximal CPET is suitable to properly express the CRF of stroke patients.

## 2 Materials and methods

### 2.1 Participants

This cross-sectional survey was conducted in accordance with the Declaration of Helsinki and was approved by the Institutional Review Board of Chang Gung Memorial Hospital, Taiwan (201600569B0) and was registered on clinical trial (NCT03960918). After the experimental procedure was explained, all enrolled patients provided written informed consent. Between June 2019 and December 2019, 37 stroke patients capable of independent ambulation who could pass a previously familiar test (free pedaling on a bicycle with a cadence of 55-65 revolutions per minute (RPM) and walking independently for 30 minute) with stable clinical presentations under optimized treatment for 6 months were enrolled. The inclusion criterion was the first primary stroke diagnosis, as confirmed by a neurologist, with admission to the rehabilitation ward of Keelung Chang Gung Memorial Hospital, Taiwan. Any participant meeting 1 or more items listed on the American College of Sports Medicine (ACSM) contraindications to exercise was excluded from the study.^[2]^ Contraindications to exercise included: acute myocardial infarction within 2 days, ongoing unstable angina, uncontrolled cardiac arrhythmia with hemodynamic compromise, active endocarditis, symptomatic severe aortic stenosis, decompensated heart failure, acute pulmonary embolism, pulmonary infarction, or deep venous thrombosis, acute myocarditis or pericarditis, acute aortic dissection, and physical disability that precludes safe and adequate testing.^[2]^

The age, sex, height, and bodyweight of eligible candidates were recorded before the CPET. Disease specifics, including stroke type (ischemic, hemorrhagic), involved side (left, right, or both), and neurological disability, according to the Brunnstrom recovery classification,^[13]^ were recorded. Medication use and comorbidity were also recorded. Health-related quality of life of these participants was evaluated by the Medical Outcomes Study Short Form-36, and physical and mental component was measured separately by the user manual.^[14]^ The mini-mental state examination was used to assess cognitive function.^[15]^

### 2.2 Cardiopulmonary exercise test

Enrolled participants performed a symptom-limited incremental CPET on a stationary bicycle (VIAsprint 150P, Cardinal Health, Basweiler, Germany) supervised by certificated physiatrists. According to the recommendation of previous literature,^[16]^ the details of the CPET were as follows: The exercise test protocol starts with a 2-minute resting phase without any body motion, then a 2-minute warm-up phase with free pedaling without any loading, followed by a ramping workload increase of 10 W/min until exhaustion.^[17]^ The test ends with a 5-minute cool-down phase under free pedaling. During the CPET, participants were asked to keep a cadence of 55 to 65 revolutions per minute. If the patient could not easily keep the hemiplegic leg on the pedal, appropriate taping was done to keep the leg anchored. The workload, VO_2_, carbon dioxide production (VCO_2_), VE, and the end-tidal partial pressures of oxygen and carbon dioxide were measured for each exhalation during CPET using an integrated auto-system (MasterScreen CPX, Cardinal-health, Germany).^[17]^ Concomitantly, variables to ensure safety during the CPET such as heart rate (HR), blood pressure, electrocardiogram, subjective rating (Borg rating of perceived exertion), and clinical signs and symptoms were recorded. The work loading during the CPET was stopped by volitional exhaustion, a pedaling cadence <40 revolutions per minute, or when the termination criteria listed in the ACSM guidelines for clinical exercise testing were met.^[2]^ The termination criteria included: ST elevation (>1.0 mm) in leads without preexisting Q waves because of prior MI (other than aVR, aVL, or V1); a drop in systolic blood pressure of >10 mm Hg, despite an increase in workload, when accompanied by other evidence of ischemia; moderate-to-severe angina; central nervous system symptoms (e.g., ataxia, dizziness, or near-syncope); signs of poor perfusion (cyanosis or pallor); and sustained ventricular tachycardia or another arrhythmia, including second- or third-degree atrioventricular block. If a participant experienced any single item listed on the ACSM termination criteria for CPET and the symptom or sign persisted for 1 minute, the test was halted, and the presence of an adverse event was recorded. Emergency intervention from the cardiologic ward would have been introduced in the case of adverse events. After test completion, the maximal values for VO_2_, VCO_2_, VE, HR, blood pressure, and workload were recorded. Maximal value was defined as the average value over the last 30 second. The following secondary parameters were calculated: respiratory exchange ratio (RER): defined as the ratio between VCO_2_ and VO_2_; VE-VCO_2_ slope: defined as the slope of the linear regression line between VE and carbon dioxide output; anaerobic threshold (AT): determined with the V-slope method, defined as the point at which a linear relationship between VCO_2_ and VO_2_ is lost^[18,19]^; oxygen uptake efficiency slope (OUES): derived from the slope of the natural logarithm plot of VE (l/min) vs VO_2_ (ml/min), which indicates the constant representing the rate at which oxygen consumption increases in response to VE, showing an estimation of ventilation efficiency with respect to VO_2_.^[6]^ Greater OUES values indicate higher ventilatory efficiency. To assess the accuracy of OUES in submaximal exercise intensity and to determine the predictive validity of the submaximal OUES, we calculated the OUES at 50%, 75%, and 100% of maximal exercise duration (OUES_50_, OUES_75_, OUES_100_).

### 2.3 Statistical analysis

For a small number of participants in the study, baseline characteristics are described using the medians with interquartile ranges (IQRs) for continuous variables and counts and percentages (%) for categorical variables. We divided the participants according to the Brunnstrom (Br.) stage of the affected lower limbs (if bilaterally affected, the stage of the worse limb was chosen), into low function (Br. stage ≤ IV) and high function (Br. stage > IV) groups as reported in Watanabe et al's study.^[20]^ Because of the small number of participants in the study, Mann-Whitney *U* test (for interval data) and Chi-square test (for categorical variables) were used to compare the participants’ characteristics as well as CPET outcomes between the 2 functional groups. Pearson correlation was used to check the relationship between AT, OUES_50_, OUES_75_, OUES_100,_ and peak oxygen consumption to validate the predictive validity of the submaximal OUES and concurrent validity of maximal OUES. The agreement between peak OUES and submaximal OUES was assessed using the Bland and Altman plot. Statistical analyses were performed using SPSS version 25.0. (IBM. Armonk, NY). A *P* value of less than.05, 2-tailed, was considered significant.

## 3 Results

### 3.1 Demographic and clinical characteristics

Thirty-seven patients with definite stroke participated in this study. Participant characteristics are summarized in Table 1. Seventeen participants (14 men, 3 women) were placed in the low function group and 20 participants (17 men, 3 women) in the high function group. Between these 2 groups, the low function group had a significantly lower average age (50.6 [IQR, 43.2-57.7] vs 62.1 [IQR, 58.0-73.8], *P* = .001) and lower Br. stage (4 [IQR, 3-4] vs 5 [IQR, 5-5], *P* < .001) than that in the high function group. There were no significant between-group differences in any other demographic variables, including stroke type, lesion site, medication, and comorbidity.

**Table 1:** Clinical characteristics of patients with post-acute stroke (n = 37).

	All (N = 37 M31/F6)	Low function (N = 17 M14/F3)	High function (N = 20 M17/F3)
Age (yrs)	58.0, [47.0-67.2]	50.6, [43.2-57.7]	62.1, [58.0-73.8]^∗^ (*P* = .001)
Height (cm)	164.5, [159.0-169.1]	166.6, [161.2-172.6]	162.8, [158.0-168.5]
Weight (kg)	69.3, [62.1-80.2]	75.4, [62.0-84.5]	66.9, [61.9-74.3]
BMI	26.1, [23.1-29.5]	26.1, [23.1-30.5]	25.8, [22.8-28.1]
Type of stroke (N)			
Ischemic	23	9	14
Hemorrhagic	14	8	6
Hemiparetic side (N)			
Right	16	7	9
Left	16	8	8
Bilateral	5	3	2
Brunnstrom stage of affected LE	5, [4-5]	4, [3-4]	5, [5-5]^∗^ (*P* < .001)
MMSE	30, [27.5-30.0]	30, [27.0-30.0]	30, [26.3-30.0]
PCS	45.9, [39.7-60.0]	47.00, [41.5-51.6]	42.9, [38.9-49.1]
MCS	42.2, [32.0-50.7]	41.3, [31.6-52.0]	42.5, [32.9-50.4]
Spasticity (MAS) 1 + = 2; 2 = 3…	0.0, [0.0-1.0]	0.5, [0.0-2.0]	0.0, [0.0-0.0]
Time post stroke (mo)	16.3 (8.6-53.9)	29.67 (11.2-77.1)	12.6 (4.3-33.9)
Medication (N,%)			
BB	16 (42%)	10 (59%)	6 (30%)
CCB	20 (54%)	9 (53%)	11 (55%)
ACEI/ARB	15 (41%)	8 (47%)	7 (35%)
Antiplatelet	23 (62%)	9 (53%)	14 (70%)
OAC	3 (8%)	1 (6%)	2 (10%)
Comorbidities (N,%)			
DM	13 (35%)	5 (28%)	8 (40%)
HTN	27 (73%)	15 (83%)	12 (60%)
Hyperlipidemia	25 (68%)	11 (61%)	14 (70%)
OSA	8 (22%)	3 (16%)	5 (25%)
Heart disease (Af/MI/HF…)	11 (30%)	4 (22%)	7 (35%)

### 3.2 CPET variables

During the CPET conducted in this study, no adverse events occurred. CPET variables are presented in Table 2. The mean maximal HR for all participants was 126 [IQR, 106-144] beats per minute (79.1 [IQR, 66.7-89.8] of predicted maximal HR) with no significant differences observed between the low and high function groups (121 [IQR, 103.5-146.5] beats per minute vs 130 [IQR, 112.3-145.0] beats per minute, *P* > .05). The mean maximal RER was 1.10 [IQR, 1.03-1.14] in the low function group and 1.11 [IQR, 1.01-1.15] in the high function group. The mean peak oxygen consumption in all the participants was 15.6 [IQR, 13.6-18.1] ml/min/kg (61.58% ± 14.75% of predicted maximal VO_2_) with no significant difference between the low and high function groups (14.6 [IQR, 12.8-19.6]ml/min/kg vs 17.0 [IQR, 15.2-17.8] ml/min/kg, *P* > .05). The average time taken for all participants to reach the maximal exercise level was 7.7 minute with no significant difference between the low and high function groups (6.9 ± 2.8 minute vs 8.5 ± 2.4 minute, *P* = .079). The AT was attained by 35 participants (35/37 = 94%), who reached a mean of 619 [IQR, 523-730] ml/min, with 57.0% [IQR, 52.0-64.0%] of peak oxygen consumption. The 2 participants who did not reach the AT level belonged to the low function group; hence, the average value of AT in the low function group was divided by 15. The mean AT in the low function group was 677 [IQR, 544-847] ml/min, with 60.0% [IQR, 50.8%-69.0%] of peak oxygen consumption and that in the high function group was 581 [IQR, 508-707] ml/min, with 56.0% [IQR, 52.0%-62.0%] of peak oxygen consumption; these differences were not significant. The mean OUES in all participants was 566.2 [IQR, 470.0-711.6] with no significant difference between the low and high function groups (579.3 [IQR, 463.2-765.4] vs 566.2 [IQR, 470.0-690.7] ml/min/kg, *P* > .05). According to the criteria for maximal exercise in the ACSM guidelines for exercise testing, the maximal HR achieved by 13 participants (13/37 = 35.1%) was >85% of the age-predicted maximal HR, and 21 (21/37 = 56.8%) achieved an RER > 1.10. This ratio is also not significantly different between the low function group and the high function group (23% vs 45%, *P* = .144, and 53% vs 60%, *P* = .549). All participants reported a Borg rating of perceived exertion >17. Hence, all participants terminated their CPET due to subjective feelings of exhaustion. The only statistically significant differences between the low and high function groups were seen in the ratio of peak HR reached in the CPET to aged-predicted maximal HR (69.1 [IQR, 63.0-89.4] vs 84.4 [IQR, 73.9-90.0], *P* = .041).

**Table 2:** Values from cardiopulmonary exercise testing of the subjects with post-acute stroke (n = 37).

	All median, [IQR]	Low function median, [IQR]	High function median, [IQR]
VO_2peak (ml/min)_	1090, [982-1169]	1093, [992-1165]	1053, [960-1265]
VO_2peak (ml/min/kg)_	15.6, [13.6-18.1]	14.6, [12.8-19.6]	17.0, [15.2-17.8]
Workload (W)	75, [70-90]	70, [60-80]	85, [70-100]
ΔVO_2peak_/ΔW	14.0, [12.8-15.2]	14.9, [13.0-17.6]	13.3, [12.1-14.0]^∗^ (*P* < .001)
AT (W)	30, [24-40]	30, [25-40]	30, [20-40]
AT (ml/min)	619, [523-730]	677, [544-847]	581, [508-707]
AT (% of VO_2peak)_	57.0, [52.0-64.0]	60.0, [50.8-69.0]	56.0, [52.0-62.0]
OUES_100_	566.2, [470.0-711.6]	579.3, [463.2-765.4]	566.2, [470.0-690.7]
R^2^ of OUES	0.91, [0.87-0.94]	0.88, [0.86-0.92]	0.92, [0.87-0.94]
% of pred. OUES	57.8, [48.5-71.2]	58.4, [45.6-70.4]	57.8, [49.8-75.8]
OUES_50_	472.7, [388.8-573.8]	513.9, [391.9-621.4]	420.0, [379.3-549.9]
OUES_75_	520.9, [451.6-664.2]	579.0, [456.6-682.2]	515.7, [439.4-625.9]
HR_max (bpm)_	126, [106-144]	121, [103.5-146.5]	130, [112.3-145.0]
% of pred. HR_max_	79.1, [66.7-89.8]	69.1, [63.0-89.4]	84.4, [73.9-90.0]^∗^ (*P* = .041)
>85% of pred. HR_max_, No.(%)	13 (35%)	4 (23%)	9 (45%)
RER	1.10, [1.02-1.14]	1.10, [1.03-1.14]	1.11, [1.01-1.15]
>1.10/<1.10, No.(%)	21/16 (57%)	9/8 (53%)	12/8 (60%)
VE-VCO_2_	35.7, [30.7-40.4]	35.3, [31.0-38.8]	36.3, [29.9-42.1]
CO (l/min)	9.0, [7.9-10.1]	9.2, [8.4-10.2]	8.8, [7.4-10.0]

### 3.3 Correlation coefficients of submaximal indices and VO_2_max

Table 3 shows the Pearson correlation coefficients between peak oxygen consumption and submaximal indices for the concurrent validity of OUES. There were statistically significant correlations between peak oxygen consumption and anaerobic threshold, OUES_50_, OUES_75_, and OUES_100_ (*r* = 0.745, 0.602, 0.726, and 0.835, respectively; *P* < .05) (Table 3).

**Table 3:** Correlation coefficients of submaximal indices and peak oxygen consumption.

	VO_2peak_	AT	OUES_50_	OUES_75_	OUES_100_	VE-VCO_2_
VO_2peak_	1	(<0.001)	(<0.001)	(<0.001)	(<0.001)	0.070
AT	0.745	1	(0.001)	(<0.001)	(<0.001)	0.173
OUES_50_	0.602	0.522	1	(<0.001)	(<0.001)	0.146
OUES_75_	0.726	0.646	0.933	1	(<0.001)	0.011
OUES_100_	0.835	0.828	0.877	0.973	1	0.004
VE-VCO_2_	−0.297	−0.235	−0.240	−0.409	−0.453	1

	VO_2max_	AT	OUES_50_	OUES_75_	OUES_100_	VE-VCO_2_
VO_2peak_	1	(0.008)	(0.005)	(<0.001)	(<0.001)	0.510
AT	0.529	1	(0.013)	(0.001)	(<0.001)	0.453
OUES_50_	0.559	0.499	1	(<0.001)	(<0.001)	0.399
OUES_75_	0.662	0.629	0.930	1	(<0.001)	0.100
OUES_100_	0.731	0.684	0.833	0.958	1	0.036
VE-VCO_2_	−0.141	−0.161	−0.180	−0.344	−0.430	1

### 3.4 Relationship between submaximal and maximal OUES for the predictive validity of the submaximal OUES

**Figure 1:**
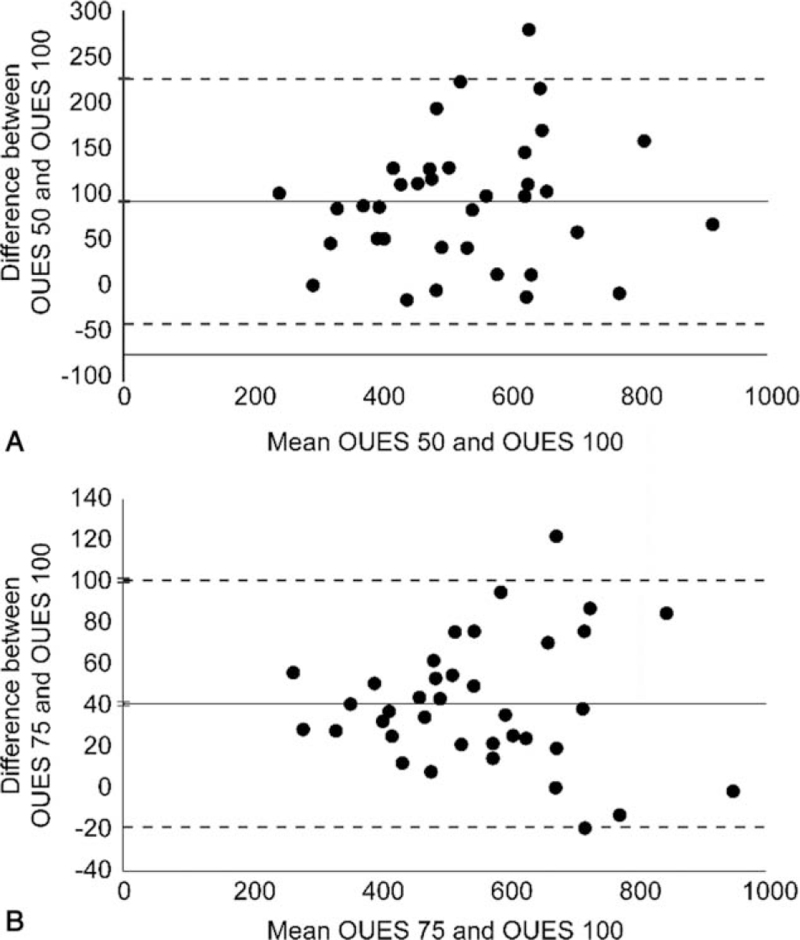
Bland-Altman plots for assessing the agreement between submaximal oxygen uptake efficiency slope (OUES) and maximal OUES of stroke patients. (A) Agreement between OUES based on 50% exercise duration (OUES_50_) and OUES from time to voluntary exhaustion (OUES_100_). (B) Agreement between OUES based on 75% exercise duration (OUES_50_) and OUES_100_. Dashed lines represent the 95% confidence limits of agreement between the 2 methods, and the horizontal solid line represents the mean difference.

OUES_50_, OUES_75_, and OUES_100_ values were 472.7 [IQR, 388.8-573.8], 520.9 [IQR, 451.6-664.2], and 566.2 [IQR, 470.0-711.6], respectively (Table 2). We considered the accuracy of the OUES_50_, OUES_75_, and OUES_100_ as being good since they demonstrated mean correlation coefficients of *r* = 0.887 ± 0.027, 0.931 ± 0.025, and 0.941 ± 0.031, respectively, when plotting log VE against VO_2_. The relationships between submaximal indices were statistically significant: OUES_50_ and OUES100 (*r* = 0.877, *P* < .001); OUES_75_ and OUES_100_ (*r* = 0.973, *P* < .001); OUES_50_ and OUES_75_ (*r* = 0.933, *P* < .001) (Table 3). Furthermore, AT showed a moderate-to-strong correlation with OUES_50_, OUES_75_, and OUES_100_ (*r* = 0.522, *P* < .001; *r* = 0.646, *P* < .001; *r* = 0.828, *P* < .001, respectively). The Bland-Altman plots in Figure 1 demonstrate the agreement between the submaximal OUES and the maximal OUES of stroke patients. Figure 1A shows the mean difference of 92.95 between OUES_50_ and OUES_100_ (95% confidence interval: −41.67-227.57). The mean difference between OUES_75_ and OUES_100_ was 41.06 (95% confidence interval: −18.64-100.77), as shown in Figure 1B. All differences, except for that of 1 individual, fell within 2 standard deviation, which demonstrates the high agreement between maximal and submaximal OUES.

## 4 Discussion

Stroke patients frequently experience varying degrees of physical disability and are thus unable to perform maximal CRF assessments, necessitating the use of submaximal assessments. Here, we investigated the effectiveness of OUES values in submaximal CRF assessments for stroke patients. We found that stroke patients in both the low and high function groups had diminished aerobic capacities. In addition, we demonstrated the validity of OUES as a measure of CRF in stroke patients showing strong correlations between OUES and peak VO_2_ and indicating the effectiveness of the OUES measure in submaximal CFR assessment.

The aerobic capacity of the stroke patients demonstrated a mean peak oxygen consumption of 15.6 [IQR, 13.6-18.1] ml/min/kg, which is only 61.58% of healthy individuals,^[21]^ a phenomenon that can be explained by primary distortion or secondary deconditioning from motor function impairment. In total, 56.8% of participants reached an RER >1.10, and all participants reported a Borg rating of perceived exertion scale >17, demonstrating that these stroke patients applied maximum effort in the CPET. However, only 35.1% of participants reached a peak heart rate ≥85% of the age-predicted maximal heart rate, without considering the influence of beta-blockers. If we account for the effect of beta-blockers (i.e., by including patients with peak heart rates >62% of the age-predicted maximal HR during the CPET), the proportion of qualifying CPETs increases to 59%.^[22]^ Furthermore, there is no statistical difference in these 3 variables between the low and high function groups. According to these points, we could postulate that the lower aerobic capacity of stroke patients is attributed to the secondary deconditioning rather than to the primary distortion. However, this may be because we recruited more chronic stroke patients in this study (e.g., average time post-stroke was 16.3 months).

In addition, we assessed the validity of the OUES, a parameter that could be derived from the submaximal CPET, using the commonly used parameters derived from the maximal CPET, such as maximal oxygen consumption, in patients with stroke. Similar to the peak oxygen consumption, the average OUES value of stroke patients was 566.2 [IQR, 470.0-711.6], only 59.66% of that of healthy individuals.^[23]^ This was even demonstrated in the group with a higher function, which was significantly lower than that of the healthy population previously reported (Table 2). The relationship between OUES and peak oxygen consumption is shown in Table 3, which shows a moderate-to-high association between these 2 submaximal and maximal variables (*r* = 0.835, *P* < .001). These results demonstrate that the OUES could be used as a measure of CRF in stroke patients. Some studies have found similar correlations between OUES and VO_2peak_ in different disease populations, mainly in heart disease,^[6,9]^ pulmonary hypertension, and pediatric fields.^[24,25]^ Meanwhile, some diseases, such as cystic fibrosis, do not show this correlation.^[26]^

This study also compared the submaximal OUES using 50% and 75% of the CPET duration (OUES_50_ and OUES_75_) with those derived from the total CPET duration (OUES_100_) to validate the predictive validity of a submaximal OUES and to demonstrate the application of OUES in the “submaximal” status. The results showed that the accuracy of OUES_50_, OUES_75_, and OUES_100_ (the correlation between log VE and VO_2_) was high. These findings indicate that the OUES from submaximal exercise duration was stable. In addition, both OUES_50_ and OUES_75_ were highly correlated with the OUES_100_ (Table 3). Moreover, a strong agreement was also shown in the Bland-Altman plots between OUES_50_ and OUES_100_ (Fig. 1A) and between OUES_75_ and OUES_100_ (Fig. 1B). Furthermore, the intra-class correlation coefficient of submaximal OUES also shows a high consistency between OUES_50_ and OUES_100_ (ICC = 0.723) and full consistency between OUES_75_ and OUES_100_ (ICC = 0.935) (Table 4). These findings indicate that, in stroke populations, the OUES can be determined using a submaximal exercise test, which could assess the actual CRF without interference from the CPET protocol and patient effort.

**Table 4:** Intra-class correlation coefficient of submaximal oxygen uptake efficiency slope.

	ICC	95% confidence interval	*P* value
OUES_100_	1	–	–
OUES_50_	0.723	−0.018–0.908	<.001
OUES_75_	0.935	0.387–0.981	<.001

Variables affecting OUES might include the effects of various systems involved in cardiovascular, respiratory, muscular, and metabolic functions.^[19]^ Owing to the heterogeneous pattern of stroke sequelae, all these components were shown to be involved in patients with stroke. For example, regarding cardiovascular function, Yperzeele et al^[27]^ reported that the autonomic dysfunction in the cardiovascular system, expressed by a decrease in HR variability, may be a precursor to adverse outcomes after a stroke episode. Regarding muscle function, the muscle strength of the hemiplegic side is obviously affected, as is the metabolic function of the muscles. Severinsen et al^[28]^ reported that the muscle type in stroke patients shifts toward a more fatigable type, and the oxidative enzymatic capacity is reduced. Regarding breathing function, Teixeira-Salmela et al^[29]^ demonstrated that the strength of the respiratory muscles is also decreased in stroke patients. These are partial mechanisms that could reduce OUES in stroke patients.

There are still other submaximal physiological outcomes that can be considered as indicators of aerobic capacity. AT, which is 1 of these submaximal variables, could be a direct indicator of aerobic capacity.^[30]^ In this study, the AT was reached in 94% of the studied subjects (the other 6% did not reach the AT value because of impaired endurance, and the CPET was terminated before the AT value was reached), which had a strong correlation with peak oxygen consumption. Its high accessibility hints that AT could be another indicator for exercise capacity, comparable with the finding of Pierre Boyne et al^[31]^ However, the AT is 57% of the measured peak VO_2_, which is in the “normal” range of the healthy population.^[19]^ If motor impairment is the main limitation for these stroke patients to achieve maximal effort in an ideal CPET, the AT level should be higher. In addition, the correlation between AT and VO_2peak_ is more fluctuating than OUES, with the correlation coefficient decreasing from 0.745 without adjustment to 0.529 after adjusting for age, sex, body mass index, and co-morbidity. Hence, using AT as an indicator for stroke patients needs further validation.

Intuitively, high functioning stroke patients had less motor impairment and should have greater aerobic capacity than low functioning patients. It has been reported that VO_2peak_ shows a tendency to be dependent on the severity of hemiparesis in the leg of stroke patients.^[32]^ In our study, there was no difference in peak oxygen consumption between the low and high function groups regardless of whether we used VO_2 peak_ or VO_2peak_/kg. The low function group in our study actually had a lower peak workload but had similar VO_2_ compared with the high function group. Although it was not statistically significant, the low function group had a lower peak workload but had a similar VO_2_ compared with the high function group. The phenomenon could be explained by the impaired motor skill or the incoordination of the motor process, which will lead to more energy expenditure.^[33]^ In fact, it could be demonstrated by the energy efficiency, expressed by oxygen consumption divided by absolute workload, which was shown to be significantly different between the 2 groups (14.9 [IQR, 13.0-17.6] vs 13.3 [IQR, 12.1-14.0], *P* < .001) (Table 2). Hence, the absolute workload could not be a good indicator of motor performance for stroke patients.

Another variable, the VE-VCO_2_ slope, can also be derived from the submaximal exercise data (Table 3). The VE-VCO_2_ slope has been used to predict heart failure prognosis.^[34]^ We found a moderate association of the VE-VCO_2_ slope with OUES and a nonsignificant association with VO_2peak_. This finding demonstrates a diminished clinical value of the VE-VCO_2_ slope in the stroke population

This study had some limitations. First, many of the recruited patients were receiving neuro-rehabilitation treatment for their chronic sequelae of stroke. Hence, these selected participants may have more motivation to engage in exercise, particularly the low function group with more motor impairment. Therefore, this group is less representative of the general stroke patient population. Second, following the tradition of our laboratory,^[35]^ we chose a natural logarithm to replace log10 for OUES calculations in our study. Thus, readers could reproduce our data by multiplying the value by 2.3 to get a generic OUES value for better interpretation. Third, unlike some studies that utilize the CPET data to calculate OUES_50_ only when the patients reach a peak HR during CPET that is >85% of the age-predicted maximal HR, we used all the patient data to calculate the OUES_50_. Because the average proportion of beta-blocker use in our study population was 42%, meeting the criteria (>85% of age-predicted HR_max_) was difficult in beta-blocker users (only 12.5% of beta-blocker users met these criteria).

In summary, our data show that the OUES is a good variable for expressing the CRF of patients with stroke because of its objectivity, validation, and reliability. It could be calculated under a submaximal exercise test regardless of the severity of motor impairment, thereby increasing its suitability for patients with stroke. However, we still cannot interchange OUES to VO_2 peak_, nor can we have a critical cut-off value to express stroke severity. Hence, the definitive formula for converting the value of OUES into the prescription of exercise is still ambiguous. Further study is needed to fill the gap of the clinical use of OUES from evaluating to prescription.

## Acknowledgments

The authors thank the Department of Physical Medicine and Rehabilitation, Chang Gung Memorial Hospital, Keelung, Taiwan, for providing the patient cohort. We also thank Professor Juei-Chao Chen for statistics consultation.

## Author contributions

**Investigation:** Sheng-Chieh Han, Chih-Chin Hsu, Shu-Chun Huang, Hsin-Yu Lin, Jong-Shyan Wang, Tieh-Cheng Fu.

**Resources:** Tieh-Cheng Fu.

**Validation:** Tieh-Cheng Fu.

**Writing – original draft:** Sheng-Chieh Han, Tieh-Cheng Fu.

**Writing – review & editing:** Sheng-Chieh Han, Chih-Chin Hsu, Shu-Chun Huang, Hsin-Yu Lin, Tieh-Cheng Fu.

## References

[1] Hsieh FI Chiou HY. Stroke: morbidity, risk factors, and care in Taiwan. J Stroke 2014;16:59–64. 24949310 10.5853/jos.2014.16.2.59 PMC4060269

[2] Riebe D Ehrman JK Liguori G Magal M. ACSM's Guidelines for Exercise Testing and Prescription. Philadelphia: Wolters Kluwer; 2018.

[3] Kelly JO Kilbreath SL Davis GM Zeman B Raymond J. Cardiorespiratory fitness and walking ability in subacute stroke patients. Arch Phys Med Rehabil 2003;84:1780–5. 14669183 10.1016/s0003-9993(03)00376-9

[4] Mackay-Lyons MJ Makrides L. Exercise capacity early after stroke. Arch Phys Med Rehabil 2002;83:1697–702. 12474172 10.1053/apmr.2002.36395

[5] Gordon NF Gulanick M Costa F. Physical activity and exercise recommendations for stroke survivors: an American Heart Association scientific statement from the Council on Clinical Cardiology, Subcommittee on Exercise, Cardiac Rehabilitation, and Prevention; the Council on Cardiovascular Nursing; the Council on Nutrition, Physical Activity, and Metabolism; and the Stroke Council. Stroke 2004;35:1230–40. 15105522 10.1161/01.STR.0000127303.19261.19

[6] Baba R Nagashima M Goto M. Oxygen uptake efficiency slope: a new index of cardiorespiratory functional reserve derived from the relation between oxygen uptake and minute ventilation during incremental exercise. J Am Coll Cardiol 1996;28:1567–72. 8917273 10.1016/s0735-1097(96)00412-3

[7] Akkerman M van Brussel M Hulzebos E Vanhees L Helders PJ Takken T. The oxygen uptake efficiency slope: what do we know? J Cardiopulm Rehabil Prev 2010;30:357–73. 20724931 10.1097/HCR.0b013e3181ebf316

[8] Onofre T Oliver N Carlos R. Oxygen uptake efficiency slope as a useful measure of cardiorespiratory fitness in morbidly obese women. PLoS One 2017;12:e0172894. 28384329 10.1371/journal.pone.0172894 PMC5383027

[9] Van Laethem C Bartunek J Goethals M Nellens P Andries E Vanderheyden M. Oxygen uptake efficiency slope, a new submaximal parameter in evaluating exercise capacity in chronic heart failure patients. Am Heart J 2005;149:175–80. 15660050 10.1016/j.ahj.2004.07.004

[10] Antoine-Jonville S Pichon A Vazir A Polkey MI Dayer MJ. Oxygen uptake efficiency slope, aerobic fitness, and V(E)-VCO2 slope in heart failure. Med Sci Sports Exerc 2012;44:428–34. 21808213 10.1249/MSS.0b013e31822f8427

[11] Bongers BC Hulzebos EH Arets BG Takken T. Validity of the oxygen uptake efficiency slope in children with cystic fibrosis and mild-to-moderate airflow obstruction. Pediatr Exerc Sci 2012;24:129–41. 22433258 10.1123/pes.24.1.129

[12] Heine M Verschuren O Kwakkel G. Validity of oxygen uptake efficiency slope in patients with multiple sclerosis. J Rehabil Med 2014;46:656–61. 24850225 10.2340/16501977-1825

[13] Medical Deartment, Harer & Row, Brunnstrom S. Movement Therapy in Hemiplegia: A Neurophysiological Approach. 1970.

[14] Health Institute, New England Medical Center, Ware JE. Institute NEMCHH. SF-36 Physical and Mental Health Summary Scales: A User's Manual. 1994.

[15] PAR, Folstein MF. MMSE: Mini-mental State Examination: User's Guide. 2001.

[16] van de Port IG Kwakkel G Wittink H. Systematic review of cardiopulmonary exercise testing post stroke: are we adhering to practice recommendations? J Rehabil Med 2015;47:881–900. 26551052 10.2340/16501977-2031

[17] Yates JS Studenski S Gollub S. Bicycle ergometry in subacute-stroke survivors: feasibility, safety, and exercise performance. J Aging Phys Act 2004;12:64–74. 15211021 10.1123/japa.12.1.64

[18] Balady GJ Arena R Sietsema K. Clinician's Guide to cardiopulmonary exercise testing in adults: a scientific statement from the American Heart Association. Circulation 2010;122:191–225. 20585013 10.1161/CIR.0b013e3181e52e69

[19] Mezzani A Agostoni P Cohen-Solal A. Standards for the use of cardiopulmonary exercise testing for the functional evaluation of cardiac patients: a report from the Exercise Physiology Section of the European Association for Cardiovascular Prevention and Rehabilitation. Eur J Cardiovasc Prev Rehabil 2009;16:249–67. 19440156 10.1097/HJR.0b013e32832914c8

[20] Watanabe H Tashiro K. Brunnstrom stages and Wallerian degenerations: a study using MRI. Tohoku J Exp Med 1992;166:471–3. 1502693 10.1620/tjem.166.471

[21] Itoh H Ajisaka R Koike A. Heart rate and blood pressure response to ramp exercise and exercise capacity in relation to age, gender, and mode of exercise in a healthy population. J Cardiol 2013;61:71–8. 23182944 10.1016/j.jjcc.2012.09.010

[22] Khan MN Pothier CE Lauer MS. Chronotropic incompetence as a predictor of death among patients with normal electrograms taking beta blockers (metoprolol or atenolol). Am J Cardiol 2005;96:1328–33. 16253608 10.1016/j.amjcard.2005.06.082

[23] Hollenberg M Tager IB. Oxygen uptake efficiency slope: an index of exercise performance and cardiopulmonary reserve requiring only submaximal exercise. J Am Coll Cardiol 2000;36:194–201. 10898434 10.1016/s0735-1097(00)00691-4

[24] Akkerman M van Brussel M Bongers BC Hulzebos EH Helders PJ Takken T. Oxygen uptake efficiency slope in healthy children. Pediatr Exerc Sci 2010;22:431–41. 20814038 10.1123/pes.22.3.431

[25] Tsai YJ Li MH Tsai WJ Tuan SH Liao TY Lin KL. Oxygen uptake efficiency slope and peak oxygen consumption predict prognosis in children with tetralogy of Fallot. Eur J Prev Cardiol 2016;23:1045–50. 26701873 10.1177/2047487315623405

[26] Williams CA Tomlinson OW Chubbock LV. The oxygen uptake efficiency slope is not a valid surrogate of aerobic fitness in cystic fibrosis. Pediatr Pulmonol 2018;53:36–42. 29064163 10.1002/ppul.23896

[27] Yperzeele L van Hooff RJ Nagels G De Smedt A De Keyser J Brouns R. Heart rate variability and baroreceptor sensitivity in acute stroke: a systematic review. Int J Stroke 2015;10:796–800. 26202709 10.1111/ijs.12573

[28] Severinsen K Dalgas U Overgaard K. Skeletal muscle fiber characteristics and oxidative capacity in hemiparetic stroke survivors. Muscle Nerve 2016;53:748–54. 26361074 10.1002/mus.24907

[29] Teixeira-Salmela LF Parreira VF Britto RR. Respiratory pressures and thoracoabdominal motion in community-dwelling chronic stroke survivors. Arch Phys Med Rehabil 2005;86:1974–8. 16213241 10.1016/j.apmr.2005.03.035

[30] Faude O Kindermann W Meyer T. Lactate threshold concepts: how valid are they? Sports Med 2009;39:469–90. 19453206 10.2165/00007256-200939060-00003

[31] Boyne P Reisman D Brian M. Ventilatory threshold may be a more specific measure of aerobic capacity than peak oxygen consumption rate in persons with stroke. Top Stroke Rehabil 2017;24:149–57. 27454553 10.1080/10749357.2016.1209831 PMC5588902

[32] Chen CK Weng MC Chen TW Huang MH. Oxygen uptake response to cycle ergometry in post-acute stroke patients with different severity of hemiparesis. Kaohsiung J Med Sci 2013;29:617–23. 24183356 10.1016/j.kjms.2013.05.004 PMC11916552

[33] VanSwearingen JM Studenski SA. Aging, motor skill, and the energy cost of walking: implications for the prevention and treatment of mobility decline in older persons. J Gerontol A Biol Sci Med Sci 2014;69:1429–36. 25182600 10.1093/gerona/glu153 PMC4271095

[34] Guazzi M Adams V Conraads V. EACPR/AHA Joint Scientific Statement. Clinical recommendations for cardiopulmonary exercise testing data assessment in specific patient populations. Eur Heart J 2012;33:2917–27. 22952138 10.1093/eurheartj/ehs221

[35] Fu TC Wang CH Hsu CC Cherng WJ Huang SC Wang JS. Suppression of cerebral hemodynamics is associated with reduced functional capacity in patients with heart failure. Am J Physiol Heart Circ Physiol 2011;300:H1545–55. 21278137 10.1152/ajpheart.00867.2010 PMC3283169

